# Influence of the COVID-19 pandemic on labor and childbirth care practices in Brazil: a cross-sectional study

**DOI:** 10.1186/s12884-023-05358-2

**Published:** 2023-02-03

**Authors:** Fabiana Ramos de Menezes, Thales Philipe Rodrigues da Silva, Mariana Santos Felisbino-Mendes, Luana Caroline dos Santos, Maria Albertina de Almeida Pereira Canastra, Maria Margarida Leitão Filipe, Mery Natali Silva Abreu, Francisco Carlos Félix Lana, Fernanda Marçal Ferreira, Alexandra Dias Moreira, Eunice Francisca Martins, Fernanda Penido Matozinhos

**Affiliations:** 1 School of Nursing, Universidade Federal de Minas Gerais, Belo Horizonte, Minas Gerais Brazil; 2 Department of Maternal and Child Nursing and Public Health, School of Nursing, Universidade Federal de Minas Gerais, Belo Horizonte, Minas Gerais Brazil; 3 Department of Nutrition, School of Nursing, Universidade Federal de Minas Gerais, Belo Horizonte, Minas Gerais Brazil; 4 Matosinhos Health Care Unit, Matosinhos, Portugal; 5 Department of Health Management, School of Nursing, Universidade Federal de Minas Gerais, Belo Horizonte, Minas Gerais Brazil; 6 School of Nursing, Universidade de São Paulo, São Paulo, São Paulo Brazil

**Keywords:** COVID-19 virus infection, Pregnancy, Childbirth, Epidemiology

## Abstract

**Background:**

It has been hypothesized that the coronavirus disease 2019 (COVID-19) pandemic may have changed the conduct of obstetric practices at the time of labor, delivery, and birth. In Brazil, many practices lacking scientific evidence are implemented in this care, which is charcaterized by excessive use of unnecessary interventions. This scenario may have been worsened by the pandemic. Thus, we analyzed the effects of the pandemic on care during prenatal care and delivery by comparing the results of two surveys (one was administered before the pandemic and the other during the pandemic) in public hospitals in Belo Horizonte - Minas Gerais (MG), Brazil.

**Methods:**

This cross-sectional and comparative study analyzed preliminary data from the study “Childbirth and breastfeeding in children of mothers infected with SARS-CoV-2”, which was conducted in three referral maternity hospitals in Belo Horizonte - MG during the pandemic in the first half of 2020 in Brazil. The final sample consisted of 1532 eligible women. These results were compared with data from 390 puerperae who gave birth in the three public hospitals in the study “Birth in Belo Horizonte: labor and birth survey”, conducted before the pandemic to investigate the changes in practices of labor and delivery care for the mother and her newborn, with or without COVID-19 infection, before and during the pandemic. In this research, “Birth in Belo Horizonte: labor and birth survey”, data collection was performed between November 2011 and March 2013 by previously trained nurses. Between study comparisons were performed using Pearson’s chi-square test, with a confidence level of 95%, and using Stata statistical program.

**Results:**

We found a significant increase in practices recommended by the World Health Organization during the pandemic including the following: diet offering (48.90 to 98.65%), non-pharmacological pain relief (43.84 to 67.57%), and breastfeeding in the newborn´s first hour of life (60.31 to 77.98%) (*p* < 0.001). We found a significant reduction of non-recommended interventions, such as routine use of episiotomy (15.73 to 2.09%), the Kristeller maneuver (16.55 to 0.94%), oxytocin infusion misused (45.55 to 28.07%), amniotomy (30.81 to 15.08%), and lithotomy position during labor (71.23 to 6.54%) (*p* < 0.001).

**Conclusion:**

Our study revealed a statistically significant increase in the proportion of use of recommended practices and a reduction in non-recommended practices during labor and delivery. However, despite advances in the establishment of World Health Organization recommended practices in labor, delivery, and birth, the predominance of interventionist and medicalized practices persists, which is worsened by events, such as the pandemic.

## Introduction

The coronavirus 2019 (COVID-19) pandemic, declared in March 2020, triggered an overload of healthcare systems worldwide, especially in localities with weak systems, such as Brazil [1, 2]. The dissemination of severe acute respiratory syndrome coronavirus 2 (SARS-CoV-2) infection  caused health services, including obstetric and neonatal care to reorganize their work processes so as to promote safe care for pregnant and postpartum women, newborns, and health professionals themselves [3, 4]. Since then, several recommendations and guidelines directed toward health professionals providing obstetric care have been published by different professional societies and international institutions [3]. These recommendations and guidelines have all sought to reduce the transmission of SARS-CoV-2 during labor, delivery, and birth in maternity hospitals [2, 5].

A study in England showed that there is no evidence to suggest that pregnant women face a greater risk of contracting SARS-CoV-2 infection than with non-pregnant women [6]. However, there is evidence to suggest that women infected with SARS-CoV-2 during pregnancy may have higher rates of maternal morbidity and mortality, including preterm delivery, preeclampsia and emergency cesarean section, neonatal morbidity, perinatal morbidity and mortality, and stillbirth, than uninfected women [7–10].

International and Brazilian recommendations and guidelines directed to health services and obstetric care providers are continuously updated by different professional societies and international institutions [4]. These recommendations and guidelines sought to reduce SARS-CoV-2 transmission during the process of labor, delivery and birth in maternity wards [5, 11–13].

Although the literature shows that pregnant women generally present with a mild or moderate clinical status when hospitalized [14], some may develop a more severe clinical status, as well as a severe COVID-19 infection, than non-pregnant women [15]. There is evidence that carriers of SARS-CoV-2 infection during pregnancy may have higher rates of maternal morbidity and mortality [7, 8], in addition to preterm delivery, pre-eclampsia and emergency caesarean section [9, 10].

In Brazil, the initial recommendations of the Brazilian Federation of Gynecology and Obstetrics Associations (FEBRASGO) aimed to reduce transmission of SARS-CoV-2 infection during care provided in the time of labor, delivery, puerperium, and abortion by implementing infection prevention and control measures [16]. The Brazilian Ministry of Health (MOH) determined the inclusion of specific population groups with a higher risk of SARS-CoV-2 infection and for developing serious clinical manifestations of COVID-19, such as pregnant women, directing differentiated strategies for such groups as priority for vaccination and care [16, 17].

Before the pandemic, the occurrence of inadequate obstetric practices during labor, delivery and birth had already been identified in Brazil, with excessive use of interventions and a high number of cesarean section [17–21]. The hospital-based survey "Birth in Brazil: National Survey into Labor and Birth", conducted in Brazil before the pandemic scenario, showed that some practices were still offered as routine care for women with usual risk pregnancy. For example, 91% of women gave birth in the lithotomic position, 36% received oxytocin infusion misused, amniotomy occurred in 39.1% of deliveries, the *Kristeller* maneuver was performed in 36.1% of deliveries, and cesarean section in 51.9% [18].

In 2018, the World Health Organization (WHO) developed a new set of recommendations for care during labor, delivery, and birth, called "*Intrapartum care for a positive childbirth experience"* [22]*.* This WHO update aimed to make the birth experience positive, with better physical, mental, and psychological outcomes for mothers, the newborn, and the family, by making mothers the center of the care provided and by including them in the conscious decision-making throughout the process [22].

In the context of the COVID-19 pandemic, evaluating the practices of delivery and birth care has become a challenge due mostly to insufficient monitoring and evaluation of the practice in maternity hospitals, but also because of the absence of a national information systems to record the actions of obstetric and neonatal care practices [23]. It is also worth noting that it is necessary to maintain continuous monitoring when managing the labor and delivery of COVID-19- positive pregnant women, and SARS-CoV-2 infection should not prevent the performance of recommended care practices [24].

In this context, the objective of the present study was to analyze the effects of the pandemic on prenatal and delivery care by comparing the results of two surveys (one was administered before the pandemic and the other during the pandemic) in public hospitals in Belo Horizonte - Minas Gerais (MG), Brazil.

## Methods

This study was an epidemiological, cross-sectional study nested in a cohort, performed using data from the study “Childbirth and breastfeeding in children of mothers infected with SARS-CoV-2”,  which was conducted with puerperae and their children born in three reference maternity hospitals in a capital city of southeastern Brazil. These data were compared with those of another  study titled “Birth in Belo Horizonte: Labor and Birth Survey”, conducted form 2011 to 2013, so as to investigate the changes in care practices in childbirth for parturient woman, with and without COVID-19 infection, and their newborns before and during the pandemic. The “Birth in Belo Horizonte:labor and birth survey” refers to a study with longitudinal design, adopted the same criteria as that the national study entitled “Birth in Brazil: National Survey into Labor and Birth” [25].

The data of the study “Childbirth and breastfeeding in children of mothers infected with SARS-CoV-2” were collected from the medical records of the hospital institutions, using a semi-structured questionnaire adapted from the survey “Birth in Belo Horizonte: Labor and Birth Survey”. We analyzed the medical records of all the women who delivered their children in the respective hospitals during the three months of high COVID-19 incidence (May, June, and July) [26, 27] in the first half of 2020 in Brazil.

The inclusion criteria for puerperal women who underwent hospital delivery for single newborns were as follows: newborns born at ≥ 22 weeks of gestation; newborns with birth weight ≥500 g; mothers admitted to one of the three selected maternity hospitals at the time of delivery and who went into labor (induced or not); birth could be either vaginal or cesarean section. Women who did not understand the Portuguese language, were indigenous, had a severe intellectual disability, were deaf, were homeless, or were convicted by a court order were excluded.

For the sample size calculation, the cohort study design was considered. A ratio of nine parturient woman from the unexposed group (without COVID-19) for each parturient woman in the exposed group (with COVID-19) was considered, given an infection rate of 10% during the epidemic period [28]. This proportion was considered for the event in the unexposed group. Furthermore, an odds ratio of 1.5 was estimated to achieve a confidence level of 95% and power of 80%. Considering the parameters mentioned above, a minimum sample of 1,893 parturient woman was estimated. 

The distribution of the number of parturient women in the respective maternity hospitals respected the proportion of the total number of births in each hospital.

Data collection was performed using the clinical records of the study population, in the months of higher incidence of COVID 19 in the year 2020, in Brazil (May, June and July) [26, 27].

Regarding the study “ Birth in Belo Horizonte: Labor and Birth Survey “, this sample comprised puerperae from seven maternity hospitals that serve the public health network and four maternity hospitals that serve the supplementary health network in Belo Horizonte, Minas Gerais, Brazil.

For comparison with the study “Childbirth and breastfeeding in children of mothers infected with SARS-CoV-2”, the same (three) hospitals from the public network were used, comprising both samples. In Brazil, these maternity hospitals are references in maternal-infant care and attend approximately 1500 deliveries per month.

The sample selection of the study “Birth in Belo Horizonte: Labor and Birth Survey” included women hospitalized at the time of delivery and their conceptuses, living or dead, with birth weight ≥ 500 g and/or gestational age ≥ 22 weeks of gestation. Women who did not understand the Portuguese language, those who were indigenous, had a severe intellectual disability, were deaf, were homeless, or convicted by a court order were excluded. The final sample included in this study comprised 390 puerperal women who delivered their children in the three public hospitals.

In this research, data collection was performed between November 2011 and March 2013 by previously trained nurses. The interviews were conducted during the womens’ hospital stay, at least six hours after delivery, which was set as the minimum time required for the puerpera’s rest [25].

In this study, the variables were selected through literature review, and were based on the 2018 update of the WHO guideline  "*Intrapartum care for a positive childbirth experience"* [22], in line with the recommendations of the National Committee for the Incorporation of Technology (CONITEC/MS) through the “National Guidelines for Assistance to Normal Childbirth”, [29]. These guidelines contain recommendations for care of the mother and newborn during labor and delivery, including variables related to recommended and non-recommedned practices during labor, delivery, and birth. Variables selected were based on the classification of obstetric practices put in place during labor and delivery that were suggested by the WHO, since this classification was adopted at data collection time, as shown in Table 1.

**Table 1:** Description of study variables

Obstetric practices during labor and delivery		
	Survey	
	**Birth in Belo Horizonte**	**Childbirth and breastfeeding in children of SARS-CoV-2 infected mothers**
**Variables**		
*Useful practices that should be encouraged*		
Providing food to parturient women	Interview	Medical Record
Allowing parturient women to have freedom to move	Interview	Medical Record
Use of Partogram (tool used to assess labor evolution)	Medical Record	Medical Record
Adopting Non-Pharmacological Methods (NPM) for pain relief	Interview	Medical Record
*Harmful or ineffective practices that must be eliminated*		
Enema	Medical Record	Medical Record
Perineal shaving	Medical Record	Medical Record
“Laying parturient women on their back with their legs raised” - position at delivery time	Interview	Medical Record
Kristeller maneuver	Interview and medical Record	Medical Record
*Practices inappropriately used at Labor and delivery *		
Amniotomy	Medical Record	Medical Record
Oxytocin infusion misused	Medical Record	Medical Record
Analgesia	Medical Record	Medical Record
Episiotomy	Medical Record	Medical Record

Estimates were presented in proportions (%), at 95% Confidence Interval (95% CI). Quantitative variables were subjected to Shapiro-Wilk test to check data asymmetry - asymmetric data were expressed as median and interquartile range (IQ). Regarding data analysis and treatment, we initially used Pearson’s Chi-square or Fisher’s Exact Test to compare the personal risk antecedents related to hospitalization, type of discharge and maternal characteristics between uninfected and infected/suspected parturients of SARS-CoV-2. It should be noted that pregnant women who were not tested for COVID-19 were also analysed. In the Brazilian scenario, due to the reduced number of tests for COVID-19, only parturients who were admitted to hospitals with signs or symptoms for COVID-19 were submitted to confirmatory tests. Therefore, the Brazilian scenario did not adopt universal testing for all parturients.

Comparisons of the evaluation of labor and birth assistance between the two studies were performed using the chi-square test or Fisher’s Exact test for independent samples and 95% confidence level. All analyses were performed using Stata software, version 16.0.

The study “Childbirth and breastfeeding in children of mothers infected with SARS-CoV-2” was approved by the Research Ethics Committee of the Federal University of Minas Gerais (under Opinion n. CAAE: 32378920.6.1001.5149). The project “Birth in Belo Horizonte: Labor and Birth Survey” was approved by the Ethics Committee of the Federal University of Minas Gerais (UFMG), (under Opinion n. CAAE - 0246.0.203.000–11).

All puerperal women and directors of each maternity hospital have signed the Free and Informed Consent Term, according to ethical guidelines described in the National Health Council Resolution n. 466, from December 12, 2012, which addresses research with human beings [30]. All procedures performed in studies involving human participants were in compliance with the ethical standards of the institutional research committee, as well as with the 1964 Helsinki declaration and with its later amendments or comparable ethical standards.

## Results

We identified 1532 eligible parturient women, of whom 46.08% were not infected, 1.83% were parturient woman infected/suspected by SARS-CoV-2, and 52.09% did not undergo evaluation of infection in the medical record. Most participants were referred for prepartum care (56.80% of non-infected women, and 42.86% of women infected and/or suspected of SARS-CoV-2) and had at least one delivery prior to the current pregnancy (62.02% of non-infected women and 64.29% of women infected and/or suspected of SARS-CoV-2). There were no differences in variables related to hospitalization, obstetric history, and type of hospital discharge according to the presence/suspicion of SARS-CoV-2 infection. Regarding personal history, a higher prevalence of asthma and chronic liver disease was identified among parturients with suspected or confirmed SARS-CoV-2 infection compared to those who were not infected (data no show).

When comparing the obstetric profile between the two surveys, it is observed that only for clinical or obstetric intercurrent events, history of caesarean section and indication for caesarean section during hospitalization there was a difference between the two surveys (Table 2).

**Table 2:** Description of variables related to obstetric profile: between study comparison of Birth in Belo Horizonte and Childbirth and breastfeeding in children of mothers infected with SARS-CoV-2

	Survey		
	Birth in Belo Horizonte	Childbirth and breastfeeding in children of SARS-CoV-2 infected mothers;	***p***-value*
**Obstetric Profile**			
**Parity**			0.409
Primiparous	38.97	38.77	
**Abortion history**			0.070
Yes	33.47	29.18	
**Clinical or obstetric complications during pregnancy**			**< 0.001**
Yes	66.67	46.02	
**History of previous caesarean section**			**< 0.001**
Yes	62.63	28.09	
**Indication for caesarean section at admission**			**0.001**
Yes	8.99	14.96	

* Pearson’s chi-square or Fisher’s exact test

Comparison of labor and birth assistance showed a greater presence of partogram and non-pharmacological methods for pain relief among women infected/suspected of SARS-CoV-2. However, when analyzing breastfeeding in the first hour of life, oxytocin infusion misused and amniotomy during labor the highest proportions were in women not infected with SARS-CoV-2 or not having covid-19 testing recorded in their medical records (Table 3).

**Table 3:** Description of variables related to delivery and birth attendance and occurrence among uninfected women and women infected or suspected of being infected with SARS-CoV-2, Belo Horizonte - MG. 2020

	No testing information in medical records	Suspected or confirmed infection		
	(***n*** = 798)	No (***n*** = 706)	Yes (***n*** = 28)	p* value
**Recommended practices during labor and delivery, and for the newborns**				
Provision of diet	99.01	98.17	100	0.445
Analgesia	31.27	27.63	15.38	0.529
Presence of a companion	93.07	95.54	90.91	0.406
Partograms	50.00	26.24	53.85	**< 0.001**
Non-pharmacological methods for pain relief	69.95	63.95	100	**0.006**
Breastfed in the first hours of life	82.76	76.09	50.33	**0.045**
**Practices not recommended during labor, delivery, and to the newborn**				
Trichotomy	0	0.84	0	0.105
Oxytocin infusion misused during labor	31.63	24.07	15.38	**0.013**
Amniotomy	17.62	12.20	7.69	**0.033**
Episiotomy	1.93	2.36	0.00	0.749
Kristeller maneuver	0.69	1.29	0.00	0.428
“Laying on the back with legs raised” position during delivery	5.57	8.01	0	0.622

* Pearson’s chi-square or Fisher’s exact

Comparisons of the results between surveys conducted before and during the pandemic indicated a statistically significant increase in recommended practices, such as: offering diet (48.90 to 98.65%), offering non-pharmacological methods for pain relief (43.84 to 67.57%), administering analgesia (14.38 to 29.47%), and breastfeeding in the first hour of life of the newborn (60.31 to 77.98%), *p* < 0.001. In conjunction with this, the presence of partogram decreased (70.21 to 39.62%, respectively, *p* < 0.001) (Table 4).

**Table 4:** Description of variables related to assistance during labor and vaginal delivery: between study comparison of Birth in Belo Horizonte and Childbirth and breastfeeding in children of mothers infected with SARS-CoV-2

	Survey		
	Birth in Belo Horizonte	Childbirth and breastfeeding in children of SARS-CoV-2 infected mothers	*p*-value*
**Recommended practices during labor, delivery, and to the newborn**			
Provision of diet			**< 0.001**
No	163(51.10)	15(1.35)	
Yes	156(48.90)	1.099(98.65)	
Non-pharmacological methods for pain relief			**< 0.001**
No	164(56.16)	310(32.43)	
Yes	128(43.84)	646(67.57)	
Presence of a companion			0.091
No	16(4.10)	59(5.90)	
Yes	374(95.90)	941(94.10)	
Partogram			**< 0.001**
No	87(29.79)	666(60.38)	
Yes	205(70.21)	437(39.62)	
Analgesia			**< 0.001**
No	250(85.62)	773(70.53)	
Yes	42(14.38)	323(29.47)	
Breastfeeding in the first hour of life			**< 0.001**
No	152(39.69)	133(22.02)	
Yes	231(60.31)	471(77.98)	
**Practices not recommended during labor, delivery, and to the newborn**			
Enema			0.771
No	292(100)	1017(99.90)	
Yes	–	1(0.10)	
Trichotomy			0.363
No	292(100)	1013(99.61)	
Yes	–	4(0.39)	
Bed rest prescription			0.166
No	260(89.04)	961(90.92)	
Yes	32(10.96)	96(9.08)	
Oxytocin infusion misused during labor			**< 0.001**
No	159(54.45)	779(71.93)	
Yes	133(45.55)	304(28.07)	
Amniotomy			**< 0.001**
No	146(69.19)	935(84.92)	
Yes	65(30.81)	166(15.08)	
Kristeller maneuver			**< 0.001**
No	237(83.45)	1050(99.06)	
Yes	47(15.73)	10(0.94)	
Position in labour “lying on your back with your legs raised”.			**< 0.001**
No	82(28.77)	987(93.46)	
Yes	203(71.23)	67(6.54)	
Episiotomy			**< 0.001**
No	241(84.27)	1029(97.91)	
Yes	45(15.73)	22(2.09)	
Elective Caesarean Section			0.179
No	67(66.34)	266(61.43)	
Yes	34(33.66)	167(38.57)	

Source: Prepared for the purpose of this study

*Pearson chi-square test; p-value in bold ≤0.05

There was a significant reduction in non-recommended interventions (p < 0.001), such as oxytocin infusion misused (45.55 to 28.07%), amniotomy (30.81 to 15.08%), Kristeller maneuver (15.73 to 0.94%), lithotomy position during delivery (71.23 to 6.54%), and routine use of episiotomy (15.73 to 2.09%). All of these variables showed statistically significant differences (*p* < 0.05) (Table 4).

## Discussion

The results of this study indicate an expressive number of missing records about the assessment of SARS-CoV-2 infection in parturient admitted to hospitals. The general recommendation for health professionals who provide care during labor and childbirth includes screening for COVID-19 of all pregnant person assisted, regardless of the symptomatological condition, with special attention to suspicious symptoms [31]. Testing for the diagnosis of infection is a relevant strategy for the protection of women, newborns and health professionals and for planning care, considering that parto of the infected population may presente nonspecific clinical conditions and considering the vulnerability of the obstetric population to COVID-19 [32]. Despite this, the result found in the presente study, in which more than half of the parturients did not have register about screening or testing in medical records, may exemplifies the limitation of financial and material resources for universal screening for COVID-19 in the obstetric population worldwide [33].

Given the possible consequences of the infection for the woman and the newborn, international guidelines point to the importance of aspects as the appropriate place for the care into healthcare faccilities, shared decision-making and the adoption of measures therapies, monitoring and appropriate care practices during labor and childbirth for women with COVID-19 infection [34, 35]. Therefore, the results found in the presente study regarding the sector to wich the patient was referred upon hospital admission, as well as the type of discharge from the hospital, did not present a significant difference between the analyzed grups. It may be inferred that the investigated hospitals were able to adapt their structure for the management of infected or suspected patients for COVID-19, since they were reference services for maternal and child healthcare.

It was observed that parturient women with suspected or confirmed infection had a higher prevalence of a history of asthma and chronic liver disease compared to those not infected. A systematic review and meta-analysis showed that the presence of at least one maternal comorbidity or a risk factor associated with COVID-19 infection during pregnancy increased the possibility of death in this population. This study further reported that among pregnant women with COVID-19 infection, most hospitalizations occurred during pregnancy, while death was most common in the puerperium period [36].

The results indicate a higher health risk profile in women with COVID-19 infection/suspicion, and confirmed a significant increase in the use of recommended practices during labor, delivery, and newborn care, with a corresponding reduction of non-recommended practices during the pandemic COVID-19 compared to the pre-pandemic study.

Regarding the comparison between the surveys before and during the pandemic, we observed an increase in the implementation of recommended practices between the two surveys. These recommended practicies included offering diet, non-pharmacological methods for pain relief, administering analgesia, and promoting breastfeeding in the first hour of life of the newborn. We further obsered a reduction in nonrecommended interventions, such as oxytocin infusion misused, amniotomy, Kristeller maneuver, lithotomy position during delivery, and routine use of episiotomy.

Although healthcare systems had to adapt to an unprecedented and uncertain public health crisis during the COVID-19 pandemic, providing fundamental care for families during labour and birth remained necessary [37, 38]. Initially, many of the health recommendations and guidelines published by different professional societies and international institutions in response to the pandemic failed to highlight the important aspects of labor and birth care, including respect, informed choice, early skin-to-skin contact with the newborn, and continuous support during labor and birth, strengthening he possibility of potentially harmful delivery experiences [38, 39].

According to the WHO guidelines for the clinical management of COVID-19, all pregnant women, including those with suspected or confirmed COVID-19 infection, should have access to high-quality, respectful, person-centered, and skilled perinatal care [13]. With this in mind, protocols for action during the pandemic were gradually adapted or modified by various international bodies and scientific societies. The most recent versions of these protocols express the importance of not separating the mother from the newborn, of skin-to-skin contact, and of encouraging breastfeeding [40].

Currently, the evidence on care during labor, delivery and newborn care also supports the importance of vaginal delivery (unless cesarean section is indicated for obstetric reasons), timely clamping of the umbilical cord, skin-to-skin contact and breastfeeding in the first hour of life, even in the context of the pandemic [13, 41].

However, a study conducted in Spanish maternity hospitals linked with the Baby-friendly hospital initiative (BFHI), which investigated women with and without COVID-19 to analyze the impact of the COVID-19 pandemic on perinatal care and breastfeeding support practices showed that women with COVID-19 suffer greater restrictions in practices compared to women without COVID-19, with lower rates of escorts during labor (84% vs 100%; *p* = 0.003), skin-to-skin contact (32% vs 52%; *p* = 0.04), co-housing (74% vs 98%; *p* < 0.001), and breastfeeding support (78% vs 94%; *p* = 0.02) [42]. Such practices were significantly less prevalent in women with COVID-19 compared to the pre-pandemic situation. Hospitals with greater commitment to implementing the BFHI practices reported higher rates of skin-to-skin contact (45.2% vs 10.5%; *p* = 0.01) and co-housing (83.9% vs 57.9%; *p* < 0.05) in women with COVID-19 [42].

Research on birth experiences conducted in the United States showed that one in three patients reported that they did not receive high-quality perinatal care during the COVID-19 pandemic [37]. Participants reported that there were many changes in the care received due to the COVID-19 pandemic [37]. Most patients experienced limitations in the presence or number of attendants during the time of labor and birth, many were separated from their NBs, and were further restricted in their options for pharmacological and non-pharmacological pain relief during the time of labor [37].

It is worth noting that a previous study conducted by Leal et al. in Brazil between the years 2011 to 2013, demonstrated a significant increase in access to appropriate technology for childbirth, with increased proportion of use of recommended practices and based on scientific evidence and reduction of non-recommended practices that are considered harmful [43]. These changes have been evolving for a long time under the premise that decision making based on scientific evidence is fundamental to the provision of health care, and this requires the transposition of research evidence in clinical practice to evidence-based clinical practice guidelines [22].

However, despite the advances in recent decades, especially those related to the implementation of the *Rede Cegonha* in 2011, an initiative whose purpose was to structure and organize maternal and child health care in Brazil, and the update of the WHO recommendations in 2018 [16, 22] with the advancement of the pandemic, the provision of services in maternity hospitals has changed. Most notably, the focus of care, usually centered on women, was changed to prioritize their safety needs [44]. Thus, the continuity of the midwifery care model, which has been shown to be essential for a positive birth experience, was discontinued or reduced in some health services [40, 44, 45], contributing to interventions without scientific evidence, such as separation of the mother from the newborn and the absence of a companion of the woman’s choice during hospitalization in labor and delivery [38, 39, 46].

It is noteworthy that the *Rede Cegonha* was inserted into the Unified Health Service, through Ordinance 1.459, of the Ministry of Health, whose purpose was to structure and organize maternal and child health care in Brazil [47]. Gradually inserted throughout Brazil, this measure proposed to create a network of care that would ensure women the right to sexual and reproductive planning and humanized care from pregnancy to puerperium, as well as guarantee children the right to safe birth, growth and healthy development [47, 48].

However, recently, the Ministry of Health established throughout the national territory the “Maternal and Child Network “ (*Rede de Atenção Materno Infantil*, in Portuguese) thus indicating the end of the *Rede Cegonha* [49]. Even after all the advances achieved by the *Rede Cegonha* with important changes in obstetric and neonatal care, including the insertion of obstetric nurses (os) in the model of care [49, 50], Maternal and Child Network came to replace the model of “humanization of labor and birth care” that had been disseminated in Brazil, by medical hegemony, removing the focus centered on the needs of women and their family, relocating to the centre of care who has the habit of intervening in the physiological processes of labor and birth putting women, newborns and their families at risk. In addition to removing obstetric nursing from this context, which has played an important role in the advancement of obstetric and neonatal care in recent decades [49, 50].

Nevertheless, the results found in the comparison of care practices before the COVID-19 pandemic and during its ocurrence, suggest that efforts to qualify childbirth care have been promising, despite the changes and challenges that the pandemic has imposed on the provisiono of healthcare facillities. The results show that public policies have had na effect on the healthcare scenario in a context that is evolving in Brazil.

It is worth noting that the process of change in labor and birth care is under development in Brazil and, in general, many advances are still needed. Despite the advances towards the establishment of the practices recommended by the WHO for labor, delivery and birth, interventionist and medicalized practice still predominates in Brazil.

With this context, there is a great need for new studies investigating the impacts of the COVID-19 pandemic in the medium and long term. Further studies are also needed in institutions with non-governmental funding, such as private hospitals, in order to direct the construction of strategies to promote positive changes in obstetric and perinatal care.

This study had limitations which should be considered, including those inherent to retrospective administrative database studies, such as missing data. Another limitation is that the sample was not fully representative of the city of Belo Horizonte and the Brazilian population. It should also be noted that data were compared using face-to-face interviews/medical records (Birth in Belo Horizonte) and medical records (Childbirth and breastfeeding in children of SARS-CoV-2 infected mothers), which may be underestimating the prevalence. Despite these potential limitations, this study followed a rigorous methodology, obtaining results corroborated by recently published literature.

The study was conducted in three maternity hospitals specialized in labor and birth assistance. In Brazil, these maternity hospitals are a reference in maternal and child care, in which the appropriate model of care for labor, delivery and birth are already well implemented, which may have directly interfered in the results of this study.

## Conclusion

The results of this study show that well implemented public policies and well-managed institutions can change the scenario of labor and birth care, promoting a significant increase in compliance with recommended practices, and a reduction in practices not recommended or deemed harmful to women and their newborns.

## Data Availability

The datasets generated and analyzed during the current study are not publicly available, as they were collected for a master’s student degree, and no provisions were made to make them publicly available in terms of participants’ consent, but are available from the corresponding author, on reasonable request.

## Abbreviations

Labor: Labor
NB: newborn
WHO: World Health Organization
MOH: Ministry of Health
PPP: Prepartum, Labor and Postpartum
ICU: Intensive Care Unit
PPE: Personal Protective Equipment
CONITEC: National Committee for Incorporation of Technology
FEBRASGO: Brazilian Federation of Gynecology and Obstetrics Associations
BFHI: Baby-friendly hospital initiative
